# Differences in the working pattern among wound, ostomy, and continence nurses with and without conducting the specified medical act: a multicenter time and motion study

**DOI:** 10.1186/s12912-016-0191-1

**Published:** 2016-11-29

**Authors:** Yukie Sakai, Tomoe Yokono, Yuko Mizokami, Hiromi Sanada, Mayumi Okuwa, Toshio Nakatani, Junko Sugama

**Affiliations:** 1 Department of Clinical Nursing, Graduate Course of Nursing Science, Division of Health Sciences, Graduate School of Medical Sciences, Kanazawa University, 5-11-80, Kodatsuno, Kanazawa-shi, Ishikawa 920-0942 Japan; 2 School of Health Sciences Faculty of Medicine, Nigata University, 2-746 Asahimaci-toori, Chuo-ku, Nigata 951-8518 Japan; 3 Department of Courses for Certified Nurses Institute for Graduate Nurses, Japanese Nursing Association, 1-2-3, Umezono, Kiyose-shi, Tokyo, 204-0024 Japan; 4 Department of Gerontological Nursing/Wound Care Management Division of Health Sciences and Nursing, Graduate School of Medicine, The University of Tokyo, 7-3-1 Hongo, Bunkyo-ku, Tokyo, 113-8654 Japan; 5 Faculty of Health Sciences, Institute of Medical, Pharmaceutical and Health Sciences, Kanazawa University, 5-11-80, Kodatsuno, Kanazawa-shi, Ishikawa 920-0942 Japan

**Keywords:** Inter-professional practice, Nursing intervention, Nursing process, Nursing workforce, Advanced nursing, Advanced practice, Wound care

## Abstract

**Background:**

To overcome the shortage of medical care delivery in the rapidly aging Japanese society, the Ministry of Health, Labour and Welfare in 2010 started to train the nurses to be able to conduct the specified medical acts. The Japanese Nursing Association conducted the educational program to train the wound, ostomy, and continence nurses for the specified medical act of wound care. However, the difference between wound, ostomy, and continence nurses who conducted the medical act and those who did not was not clear. The aim of this study was to determine how trained wound, ostomy, and continence nurses spend their time during their entire shift in an acute hospital setting.

**Methods:**

In this prospective observational study, we selected those wound, ostomy, and continence nurses who received advanced training in the wound management program (T-WN) in 2011–2012. Wound, ostomy, and continence nurses who did not receive the training (N-WN) were also recruited as controls. We conducted a time and motion study during subject's day shifts for 1 week. We calculated the time spent on tasks based on a task classification code that was created to facilitate a two-group comparison.

**Results:**

Six T-WNs and five N-WNs were our analysis subjects. T-WNs spent significantly more time on direct care than did N-WNs (*p* = 0.00). Moreover, in the sub-categories s of direct care, T-WN spent significantly more time on “treatment” than did N-WN (*p* = 0.01). T-WN spent significantly more time on treatment with (*p* = 0.03) or without (*p* = 0.01) physicians than did N-WN. In the treatment activities, T-WN performed significantly more time on foot care (*p* = 0.01), wound cleansing (*p* = 0.01) and conservative sharp wound debridement (*p* = 0.01) than did N-WN. Frequencies of direct care interventions for the patients was significantly different between T-WN and N-WN (*p* = 0.04).

**Conclusions:**

T-WNs frequently engaged in direct care provided treatment for patients with chronic wounds.

## Background

It has been estimated that in Japan, the percentage of the population aged ≥65 years will reach 30.3% by 2025 [1]. Because of the sudden surge in medical care needs, a deficiency in medical care has been predicted for the super aging population. In particular, shortage of physicians [2] is a significant contributor to this problem. It may be said that securing staff for medical care is an urgent problem for the fastest aging society in the world.

Nurse practitioners manage medical care in the legal model in the United States of America, European countries, and Asian countries. The utility of NP is reported from these countries. The Japanese government announced that a group of nurses who performed medical acts under a physician's direction would be formed as a solution for the aging society; these positions were named NPs. In other words, this meant that NPs could be a suitable solution for the Japanese situation. The Ministry of Health, Labour and Welfare selected 147 medical acts (called specified medical acts) which could be performed by nurses. A specified medical act included related wound treatment activities like debridement of a pressure ulcer (PU), suturing of a wound, and selection of external applications.

In response to this, therapeutic intervention for the chronic wounds would have likely been handled by the Wound, Ostomy, and Continence nurses (WOCNs). WOCNs were certified nurses who have special knowledge and skills in the wound, ostomy, and continence fields from the Japan Nursing Association after they completed an education course [3]. In Japan, WOCNs were expected to perform specialized evidence-based wound and skin care in all healthcare settings, including hospitals, long-term care facilities, and outpatient clinics. They also took the initiative in the management of patients with acute and chronic wounds, particularly PUs. WOCNs would not legally be permitted to perform invasive activities like sharp wound debridement. Therefore, they focused on wound prevention. WOCNs have a dilemma in that they cannot treat wounds in a timely manner although they have the knowledge and skills. As for the new system that the government has developed, WOCNs would be allowed to provide therapeutic intervention for wounds.

NPs deliver safe and high-quality medical services to individuals worldwide. They have delivered a wide range of medical services that include acute medical care and care for chronic conditions and pediatric, gerontology, and women’s health [4]. Although systems and medical situations differ among countries, NPs have legal autonomy to perform medical examinations, order examinations, diagnose symptoms, and prescribe treatments. There is a consensus regarding their role of autonomy [5–7]. NPs spend 40%–70% [8–10] of their time on direct patient care, and their working pattern was similar to those of physicians. NPs spent more time at the bedside than physicians; they also had dedicated communications with the medical staff and delivered care to patients [11]. Collaboration with NPs, including sharing patient’s information with NPs, receiving advices from NP, and receiving education about medical knowledge and skills from NPs, enhanced staff’s practice [12–14]. NPs play a role in enhancing inter-professional work [8].

The wound management nurse practitioner, tissue viability nurse, and WOCNs, which were termed as an advanced practice nurse (APN) could be utilized for wound management. Timely and appropriate wound bed preparation [15, 16] coordination of the care environment [17], psychological support [18], and long-term follow-up systems [19] are important for the promotion of early wound closure and the improvement of the well-being of patients with chronic wounds. APNs treat patients with wounds by conducting wound bed preparation, which would include wound debridement and prescription of external applications and antibiotics [20].

APNs conduct sharp wound debridement and prescribe external applications and antibiotics. They also establish the treatment environment through education of the medical staff as well as topical wound management [20]. Furthermore, the wound care center of a hospital base and the outpatient base are increasing both in Europe and the US [19]. There are also places where patients with wounds can be followed-up for appropriate treatment by wound specialists. Because of the shortage of physicians who specialize in wound care, team approaches cannot appropriately treat all patients with wounds in Japan.

Utilization of WOCNs who performed specific medical acts benefits patients with chronic wounds. WOCNs have currently been engaged in highly cost-effective PU management [21]. Trained WOCNs (T-WNs) may perform conservative sharp wound debridement on PUs with significant cost effectiveness [22]. This cost effectiveness has also been demonstrated for high-grade ultrasound- and thermography-driven PU assessments and conservative wound debridement/negative pressure wound therapy (NPWT) recovery protocols that have been utilized by T-WNs [23]. These data indicated that Japanese WOCNs can perform high quality advanced wound management.

**Fig. 1:**
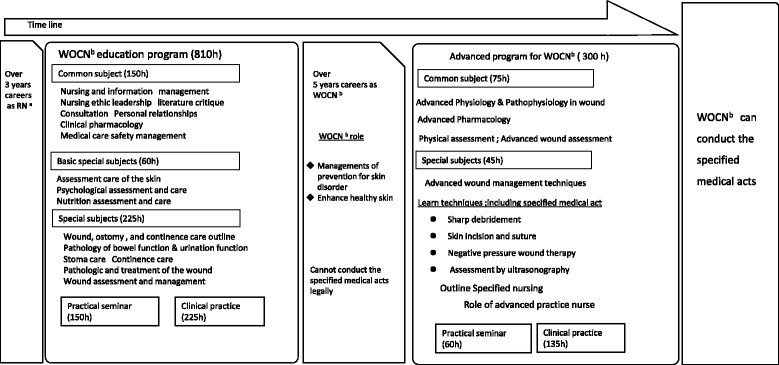
The process to become a WOCN conducting specified medical act in Japan. ^a^ Registered nurse, ^b^ Wound, ostomy and continence nurse. It is necessary to complete the advanced course and over five years WOCN experience and so WOCN can conduct the specified medical act

The training of a nurse who conducts specific medical acts is Japan’s original system cannot be extrapolated to the working pattern of physicians and NPs. The law has secured an autonomy for NPs that is similar to that for physicians. In Japan, WOCNs do not have the authority to prescribe debridement and medicines for external application. T-WNs can perform this medical act; however, it remains within the physician's range of indications. The specific acts that T-WNs are permitted to perform are limited. There is also limited information on the working pattern, i.e., the duration spent in wound bed preparation by NPs. Figure 1 shows a summary of the training received by WOCNs for specified medical act.

Evaluation of a new medical system for nurses utilizing specific medical acts is very important for the understanding their contributions to the healthcare system. Donabedian explained the quality of medical care using the framework of three domains: structures, process, and outcomes [24]. Good processes produce good outcomes. Education about specific acts is an approach for structural changes. It may affect process changes. However, it is not clear how the process of T-WN changes after having received the education. The aim of this study was to clarify the process changes in specific acts through education.

## Methods

This was a prospective, observational study as well as a time and motion study.

### Setting and participants

We conducted a prospective, observational time and motion study in acute hospitals from April 2013 to April 2014. The subjects of our study were the those T-WNs drawn from the 12 nurses who had completed the Japanese Nursing Association (JNA) Project for the Development of WOC Nurse Specialists Research Trial training course from 2011 to 2012; the participants also belonged to a medical establishment and were also registered at that establishment as part of the “Specialist Duties Project Trial.” This training lasted for 240 h and included units in pathophysiology, pharmacology, and physical assessment, along with a practicum in advanced wound management.

Here we compared T-WNs with the non-trained WOCNs (N-WNs) as controls to clearly differentiate between T-WNs and N-WNs activities.

We omitted bias by matching T-WNs and N-WNs according to institutional properties (hospital functions, bed capacity, and hospital departments including cosmetic surgery, dermatology, and vascular surgery) and WOCNs’ experience. The eligible WOCNs were introduced by a director of department of courses for certified nurses in JNA.

### Data collection

We conducted a time and motion study in which the observer of this study accompanied WOCNs during their day shifts for 1 week. Because all subjects had the weekend (2 days) off, we observed them for 5 consecutive days. We could accurately assess their complicated duties. WOCNs performed many duties at the same time or sequentially and were very busy. Self-reporting was one method of measuring work activities. However, this method might be a burden for WOCNs and it might be difficult for them to correctly remember all of their activities after the day shift. Therefore, we chose a time-and-motion study.

### Validity of the observer

One researcher who had completed the WOCN education program and had 9 years of experience as a WOCN conducted the time-and-motion study. An observer was left alone to collect consistent and correct data. The total number of activities recorded by self-reporting were significantly fewer than those recorded by the time-and motion-study measurement [25]. This result indicated that the participants were not very accurate when self-reporting their activities alone. Self-reporting might be difficult because they were very busy. Therefore, accurate consistent data could be collected in this study using an observer.

### Activity categories code

We created an activity categories code that was used in our observational study according to the following procedure:

1. Relevant categories were selected by referencing the category codes used in time studies conducted in prior studies with physicians and NPs [9, 26, 27].
2. Activities were selected and classified following a pilot study with T-WNs at a baseline institution.
3. At a second institution, another pilot study was conducted utilizing the activity category codes.

The activity category codes comprised categories (4 entries), sub-categories (22 entries), and activities (107 entries).

The definition of the categories were as follows: (1) direct care included all tasks that involved direct patient care; (2) indirect care referred to the indirect provision of patient care, including record keeping, data collection, and providing explanations to healthcare providers; and (3) administrative activities were those that were necessary to perform other tasks, including meetings, scheduling, and intra-hospital training; (4) Others included cleanup, movement, rest time. Our task documentation recorded the number of times that a task for a patient was initiated and concluded as well as providing a description of the task performed. In the cases of direct care, tasks were combined and executed in succession. For example, during a dressing change, WOCNs would perform a physical assessment and wound treatment while providing professional medical counsel to patients and their families. In other words, “treatment” which was a sub-category of direct care also included education and evaluation of patients. To clarify which activities were performed in relation to chronic wounds, we classified the activities based on whether or not they accompanied treatments such as dressing exchanges. In the case of the previous example, we calculated that WOCNs spent their time on the subcategory of direct care “treatment.” Therefore, other sub-categories of direct care did not include treatments such as dressing changes.

### Institution and subject characteristics

We collected data on characteristics that could influence our subjects’ work. For the institutions, data collection concerned hospital type, bed capacity, bed occupancy rate, mean number of outpatients in 1 day, mean days of hospitalization, number of WOCNs, and safety administration. We collected data on our subjects regarding their age, nursing experience, and years of experience as a WOCN at their institution.

### Analysis

We calculated the activity times of the participants by categories and sub-categories in accordance with the activity classifications for 1 week. We categorized the total frequency of patient interventions by problem type, such as PUs, diabetic foot ulcers (DFUs), venous leg ulcers (VLUs), surgical-site infections dehiscence wounds, skin problems, stomas, and incontinence. Because these activities were performed simultaneously or continuously between one patient and the next, it was difficult to subdivide them based on time. We counted the frequency of specified medical acts that were included in the sub-category and were performed in the sub-categories of direct care. Specified medical acts included wound debridement, NPWT, and the selection of topical treatments such as topical agents  or wound dressings. We calculated the time spent according to the location of their activities. Places included wards, outpatient clinics, outside the hospital, and at their desk (desk work; the place where subjects normally perform stationary activities). Using descriptive statistics, the interval scales that conformed to a normal distribution were mean (SD) by group otherwise, a median (range) was calculated. In our comparison of T-WNs and N-WNs, the mean was tested via the *t*-test and the median was tested via the Mann–Whitney *U* test. Statistical significance was considered at a *p* value of <0.05. We used the statistical analysis package SPSS for Windows ver. 23.

## Results

### Overview of subjects

**Fig. 2:**
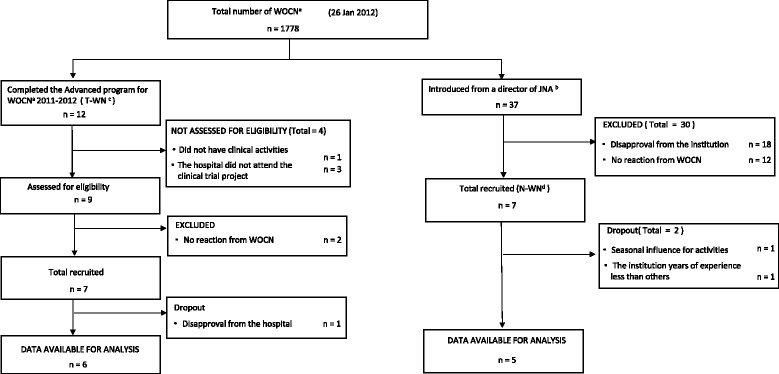
Flowchart of participants. ^a^ wound, ostomy, and continence nurse, ^b^ Japanese Nursing Association, ^c^ trained wound, ostomy, and continence nurse, ^d^non-trained wound, ostomy, and continence nurse

Out of a pool of 12 T-WNs, six participated in the study and were the subjects of our study. Thirty-seven N-WNs were chosen by snowball sampling and of these, seven participated in this study; however, two were ultimately excluded and a total of five analysis subjects were included (Fig. 2). One individual was excluded because the individual claimed that because of seasonal influence, the tasks were atypical. The other individual had 1 year and 6 months of experience at the participant’s institution of employment, had an evidently different working style compared with our other subjects, and therefore, was marked as an exception. A summary of our analysis subjects can be found in Table 1. Among our T-WNs, the median years of experience of a registered nurse was 24 (range 18–28), with a mean of 13 (SD2.6) years as a WOCN at their institution of employment. Two individuals belonged to university hospitals, which had the mean bed capacity of 685 beds (SD311.0), the mean outpatients daily of 1,505.2 (SD525.4), and the median hospitalization days of 12.1 days (range 11.9–14.1 days). Among N-WNs, the median years of experience of a registered nurse was 24 years (range 14–25), and their mean years of experience as a WOCN at their institution of employment was 9 years (SD3.7). One individual belonged to a university hospital; the mean bed capacity was 606.0 beds (SD189.8), there was the mean outpatients daily of 1,419.9 (SD425.3), and the median hospitalization was 12.6 days (range 12.2–15.2). The hospitalization was significantly shorter between the institutions of T-WNs and N-WNs (*p* = 0.05).

**Table 1:** Participants’ demographic characteristics

			T-WN^a^		N-WN^b^		
			*n* = 6		*n* = 5		*p* value
WOCN properties							
Nursing years of experience	median	range	24.0	18–28	24.0	14–25	0.70^c^
WOCN^e^ years of experience	mean	SD	13.0	2.6	9.0	3.7	0.70^d^
Institution properties							
Type of hospital							
University hospital			2		1		
Community-based hospital			4		4		
Bed capacity	mean	SD	685.0	311.0	606.0	189.8	0.78^d^
Bed occupancy rate	mean	SD	85.7	5.9	84.6	8.5	0.93^d^
Outpatients one day	mean	SD	1505.2	525.4	1419.9	425.3	0.78^d^
Hospitalization days	median	range	12.1	11.9–14.1	12.6	12.2–15.2	0.05^c^

^a^Trained Wound,Ostomy and Continence Nurse, ^b^Non-trained Wound,Ostomy and Continence Nurse, ^c^Mann-Whitney *U* test, ^d^ *t*- test, ^e^Wound,Ostomy and Continence Nurse

### Results of the time and motion study

#### Incident occurrence

There were no incidents that occurred as a result of any specialized acts performed by our subjects during the study period.

#### Time of each categories

Table 2 shows the time of each categories. The total observation time of our time and motion study was 28,837 min. Among T-WNs, 1,164.2 min per person was spent on direct care (44.6%); 336.5 min per person was spent on indirect care (12.8%); and 629.7 min per person was spent on administrative tasks (24.1%). Among N-WNs, 531.6 min per person was spent on direct care (20.3%); 584.6 min per person, per week was spent on indirect care (22.3%); and 997.2 min per person was spent on administrative activities (37.7%). T-WNs spent a significantly greater amount of time on direct care than that did by N-WNs (*p* = 0.00).

**Table 2:** Duration of time spent in each categories

				T-WN^a^			N-WN^b^			
				*n* = 6			*n* = 5			*p* value^c^
				min./week			min./week			
Total observation	mean	SD		2615.8	144.8		2628.4	96.2		0.87
Direct care	mean	SD	%	1164.2	223.5	44.6	531.6	161.2	20.3	0.00
Indirect care	mean	SD	%	336.5	146.9	12.8	584.6	233.5	22.3	0.06
Administration	mean	SD	%	629.7	96.5	24.1	997.2	398.7	37.7	0.11
Others	mean	SD	%	485.5	82.6	18.5	515.0	50.9	19.6	0.51

^a^Trained Wound, Ostomy and Continence Nurse, ^b^Non-trained Wound, Ostomy and Continence Nurse, ^c^ *t*-test

#### Time of sub-categories

Table 3 shows the amount of time spent on each task by the sub-categories. T-WNs spent the mean of 759.3 min (SD317.1) per person on treatment. N-WNs spent the mean of 226.2 min (SD 142.4) per person on treatment. The time spent on treatment by T-WNs was significantly greater amount of time than that did by N-WNs (*p* = 0.01). There were no statistically significant differences among the other sub-categories.

**Table 3:** Duration of time spent on sub-categories

			T-WN^a^		N-WN^b^		
			*n* = 6		*n* = 5		*p* value
			min./week		min./week		
Direct care							
Consultation	median	range	0.0	0–18	0.0	0–21	0.82^c^
Education	median	range	20.0	0–48	0.0	0–14	0.07^c^
Examination	median	range	4.0	0–66	0.0	0–0	0.08^c^
Treatment	mean	SD	759.3	317.1	226.2	142.4	0.01^d^
Cooperation	median	range	66.5	0–117	0.00	0–45	0.05^c^
Evaluation	mean	SD	70.3	45.4	77.2	59.1	0.83^d^
Stoma	mean	SD	210.5	114.5	192	174.8	0.84^d^
PU^e^ prevention	median	range	0.0	0–22	12	0–34	0.18^c^
Incontinence	median	range	0.0	0–11	0.0	0–8	1.00^c^
Other	median	range	11.0	3–22	1.0	0–8	0.06^c^
Indirect care							
Information	mean	SD	56.5	45.8	65.6	28.4	0.71^d^
Record	mean	SD	122.7	53.7	305.6	193.0	0.10^d^
Treatment	mean	SD	1.5	3.7	0.0	0.0	0.39^d^
Consultation	mean	SD	68.8	44.1	100.4	46.3	0.28^d^
Report	median	range	14.5	0–27	6.00	0–79	0.46^c^
Coordination	mean	SD	74.2	40.4	93.60	61.3	0.54^d^
Administration							
Education	mean	SD	45.5	32.9	93.4	63.4	0.14^d^
PU^e^ management	mean	SD	142.7	70.7	359.4	308.5	0.13^d^
Consultation	mean	SD	58.5	78.5	21.2	15.2	0.33^d^
Duties	mean	SD	363.0	197.7	523.2	249.2	0.26^d^
Training	median	range	0.0	0–81	0.0	0–0	0.18^c^

^a^Trained Wound,Ostomy and Continence Nurse, ^b^Non-trained Wound,Ostomy and Continence Nurse, ^c^Mann-Whitney *U* test, ^d^ *t* - test, ^e^pressure ulcer

#### Frequency of direct care interventions for the patients

Table 4 shows the frequency of interventions for patients who received some form of direct care. T- WN performed direct care to the mean73.2 individuals (SD15.8) per person and N-WNs did to the mean 43.0 individuals (SD 7.5) per person. The total for T-WNs was significantly larger than that for N-WNs (*p* = 0.04). In the breakdown of the totals, T-WNs performed direct care to the mean of 12.6 individuals (SD 11.9) of patients with DFUs. N-WNs did not care for patients with DFUs or VLUs.

**Table 4:** Frequency of interventions to the patients on direct care

			T-WN^a^		N-WN^b^		
			*n* = 6		*n* = 5		*p* value
			n/week		n/week		
Total patient	mean	SD	73.2	15.8	43.0	7.5	0.04^c^
PU^e^	mean	SD	16.5	6.8	18.8	7.5	0.61^c^
Diabetic foot ulcer	mean	SD	12.6	11.9	0.0	0.0	0.04^c^
Venus leg ulcer	mean	SD	1.8	2.7	0.0	0.0	0.17^c^
Peripheral arterial disease	median	range	4.0	0–17	0.0	0–8	0.13^d^
Surgical site infection wound dehiscence	median	range	6.0	1–11	0.0	0–8	0.09^d^
Other ulcer	mean	SD	12.3	10.3	3.0	2.3	0.08^c^
Skin disorder	mean	SD	4.0	4.1	5.0	3.5	0.94^c^
Stoma	mean	SD	13.3	3.2	11.8	9.1	0.88^c^
Incontinence	median	range	0.0	0–2	0.0	0–1	0.56^d^

^a^Trained Wound,Ostomy and Continence Nurse, ^b^Non-trained Wound,Ostomy and Continence Nurse, ^c^ *t*-test, ^d^Mann-Whitney *U* test, ^e^pressure ulcer

#### Time for sub-categories of direct care treatment with and without a physician

Table 5 shows the time for subcategories of direct care treatment with and without a physician. We compared the amount of time spent on the sub-categories of direct care treatment with and without a physician. When a physician was present, the mean time spent on the sub-categories by T-WNs was 384.8 min (SD181.7) per person, N-WNs spent 137.4 min per person. When a physician was not present, the mean time spent on the sub-categories by T-WNs was 377.3 min (SD 177.7) per person, and N-WNs spent the mean 90.6 min (SD 91.0) per person. The time difference was significantly higher for T-WNs than for N-WNs both when a physician was present (*p* = 0.03) and not present (*p* = 0.01).

**Table 5:** Duration of time spent on sub-category treatment of direct care with and without physician

			T-WN^a^		N-WN^b^		
			*n* = 6		*n* = 5		*p* value^c^
			min./week		min./week		
Treatment with physician	mean	SD	384.8	181.7	137.4	125.2	0.03
Treatment without physician	mean	SD	377.3	177.7	90.6	91.0	0.01

^a^Trained Wound,Ostomy and Continence Nurse, ^b^Non-trained Wound,Ostomy and Continence Nurse, ^c^ *t*-test

#### Frequency of specified medical acts on sub-category treatment

Table 6 shows the frequency of specified medical acts on sub-category of treatment.

**Table 6:** Frequency of specified medical acts on sub-category treatment

			T-WN^a^		N-WN^b^		
			*n* = 6		*n* = 5		*p* value
			n/week		n/week		
Foot care	mean	SD	9.0	5.3	0.0	0.0	0.01^c^
Conservative sharp wound debridement	median	range	1.0	0–7	0.0	0–0	0.01^c^
Wound cleansing	mean	SD	16.0	5.8	5.0	5.3	0.01^c^
Curettage	median	range	4.0	0–16	0.0	0–0	0.03^d^
Incise	median	range	0.5	0–3	0.0	0–0	0.08^d^
Determining of topical agent	mean	SD	11.1	5.2	3.8	4.4	0.04^c^
Determining of wound dressing	mean	SD	13.5	1.8	8.2	6.8	0.16^c^
Performing NPWT^e^	median	range	1.5	0–9	0.0	0–2	0.37^d^

^a^Trained Wound,Ostomy and Continence Nurse, ^b^Non-trained Wound,Ostomy and Continence Nurse, ^c^ *t*- test, ^d^Mann-Whitney *U* test, ^e^Negative pressure wound therapy

We compared the frequency of specified medical acts within direct care of treatment. T-WNs performed in foot care (*p* = 0.01), conservative sharp wound debridement (*p* = 0.01), wound cleansing (*p* = 0.01), curettage (*p* = 0.03), and determining of topical agent (*p* = 0.04) at a significantly greater frequency than N-WNs.

#### Place of practice

Table 7 shows where they performed their activities. We compared the practice places of T-WNs and N-WNs. T-WNs spent the mean of 862.8 min (SD171.1) per person in the inpatient department. N-WNs spent 654.8 min (SD 676.0) per person. T-WNs worked in inpatient department for a significantly longer time period than N-WNs (*p* = 0.04). For desk work, T-WNs spent the mean of 630.3 min (SD 341.6) per person, whereas N-WNs spent the mean of 1,132.6 min (SD 1,205.0) per person. N-WNs spent significantly more time at desk than that did by T-WNs (*p* = 0.03).

**Table 7:** Duration of time spent on participant's activities by place

			T-WN^a^		N-WN^b^		
			*n* = 6		*n* = 5		*p* value
			min./week		min./week		
Inpatient department	mean	SD	862.8	171.1	654.8	676.0	0.04^c^
Outpatient department	mean	SD	640.0	283.1	331.8	297.0	0.08^c^
Desk	mean	SD	630.3	341.6	1132.6	1205.0	0.03^c^
Other institution	median	range	0.0	0–251	0.0	0–0	0.39^d^
Not applicable	median	range	452.0	297–712	588.0	274–598	0.65^d^

^a^Trained Wound,Ostomy and Continence Nurse, ^b^Non-trained Wound,Ostomy and Continence Nurse, ^c^ *t*- test, ^d^Mann-Whitney *U* test

## Discussion

Our study was the first time and motion study that has been conducted to focus on the activity time of WOCNs. By conducting a time and motion study using one researcher who was a WOCN, we could collect accurate data about work activities in this study. To clarify the activities performed by T-WNs, we held a time study of T-WNs and N-WNs and compared their activities. The results showed that T-WNs frequently provided direct care, engaged in medical intervention, and performed specialized medical acts in patients with chronic wounds. This suggested that T-WNs could contribute to early stage wound recovery and improvement of well-being in patients by taking part in planning with the chronic wound team.

The time that T-WNs spent on direct care was 44.6% compared with the 20.3% time that N-WNs spent. This result was similar to the 46.0% observed for NPs [8]. Based on NP independence and their ability to engage in medical acts, a consensus has been reached regarding their role in clinical practice [28]. Autonomy could be an important element in maintaining the time that nurses can spend on direct care. In the case of the N-WN group, there was a difference of 37.7% in the time spent on administrative tasks compared with that spent on both direct (20.3%) and indirect (22.3%) care. As the managers of PUs, WOCNs have a role in the implementation of guideline-informed [29] PU prevention strategies in their institutions. They have been tasked with creating treatment programs for high-risk PU patients, reviewing PU care results and records on a weekly basis, and calculating the predicted occurrence and prevalence rates of PUs on a monthly basis for institutional quality evaluations. Therefore, WOCNs spent a great deal of time on desk work.

Furthermore, 44.6% of the time that T-WNs spent on direct care was similar to that spent by physicians, who spend 40%–50% of their time on direct care tasks [30]. T-WNs spent significantly more time on direct care treatment than N-WNs. T-WNs provided medical treatment both when accompanied by a physician and independently. By providing medical care in the presence of a physician, T-WNs are able to confirm the patient’s medical plan. With a focus on medical intervention, T-WNs can work on the same level as that of the physicians. This suggested that by allocating roles, it may be possible to efficiently improve patient outcomes.

The most common sub-category in direct care was wound treatment, and its most common invasive specified medical act was conservative sharp wound debridement. Considering that no complications occurred during the study period, this result suggested that T-WNs were capable of conducting medical interventions while maintaining the patient’s safety. This was related to the frequency of care intervention for PUs and DFUs, which are defined by necrotic tissue. Conservative sharp wound debridement by T-WNs has been shown to be cost-effective [22], and it has been anticipated that T-WNs proactively practice specialized medical acts on patients with chronic wounds.

It can be expected that in the area of chronic wounds, patient outcomes can be improved and safe medical care can be provided via T-WN-executed medical interventions. In a study on inter-professional, interdisciplinary team approach, patient outcomes showed improvement [31]. The importance of team care has been established for chronic wounds [32]. The safety and ease of the practice of specialized acts based on coordination and wound management techniques developed by WOCNs has been established in a qualitative analysis of T-WNs [33]. The qualitative analysis of T-WN revealed that T-WN conducted low invasive and high safety specified medical acts based on wound management techniques and coordination skills that they developed as WOCN [33]. T-WNs can provide support to medical staffs regarding the concerns and doubts related to the daily activities regarding wounds. This can be established as a safe practice in the medical profession.

There were four limitations to our study. The first was the week of day shifts that constituted the observational period for our subjects in the time and motion study and the classification of tasks during those day shifts. There was a possibility that indirect care responsibilities, such as administrative tasks, came up after the day-shift hours. The second was that the institutions of the N-WNs and T-WNs groups were not matched. There was a possibility that the skin problems of the care recipients may have been different. The third was that this study occurred 2 years after the completion of the training program. T-WNs in their first year may still be in the stage of testing out their expanded roles and may not be able to sufficiently demonstrate their skills. The fourth was that our sample size was small. Our study was conducted during the 2nd year following the completion of the training program, and there were a total of 12 T-WNs. Only six participated, and there may be limitations in applying our findings to the entire population. However, our findings were based on direct observation by one WOCN during the day shift, which produced more reliable results than interval observations or self-reporting. Our future challenge will be to conduct research that would highlight what type of patient outcomes may be produced by WOCN practices.

## Conclusions

We conducted a time-and-motion study to ascertain the activities of T-WNs. Our results led us to the following findings:

1. T-WNs spent 44.6% of their time on direct care, and within that, 759.3 min per person, per week were devoted to wound treatment.
2. T-WNs provided direct care to 73.2 patients per week on the mean and in particular, to patients with chronic wounds.
3. The time that T-WNs spent on direct care treatment was significantly greater than that spent by N-WNs both when accompanying and not accompanying a physician.
4. Within the direct care treatments, foot care, wound cleansing, conservative sharp wound debridement, curettage wound bed preparation, and topical agent selection were performed. During the study period, there were no complications that arose because of implementation of specified medical acts.

Based on the above findings, it appeared that T-WNs were capable of providing medical intervention and wound bed preparation to patients with chronic wounds. Hereafter, it may be anticipated that T-WNs may be able to contribute to improvement in the well-being of chronic wound patients.

## Abbreviations

APN: Advanced practice nurse
DFU: Diabetic foot ulcers
NP: Nurse practitioner
NPWT: Negative pressure wound therapy
N-WN: Non trained Wound, Ostomy, and Continence nurses
PU: Pressure ulcer
T-WN: Trained Wound, Ostomy, and Continence nurses
VLU: Venous leg ulcers
WOCN: Wound, Ostomy, and Continence nurses
